# Supplementary material for: Swin-HSSAM: A green coffee bean grading method by Swin transformer
Source: PLoS One. 2025 May 14;20(5):e0322198. doi: 10.1371/journal.pone.0322198 (PMC12077702; doi:10.1371/journal.pone.0322198)
Supplement: Attachment — Submitted filename: Response to Reviewers.docx [file pone.0322198.s002.docx]

Response to Reviewers

Reviewer #1:

1. Lack of broader data validation:
   Although the paper uses a proprietary dataset and one public dataset for validation, the diversity and scale of the datasets are relatively limited.

Response：We have searched for a large number of datasets on the web, but the datasets that meet the requirements for the topic of our project on raw coffee bean identification are quite sparse, and the public and proprietary datasets used in the current article are already the larger datasets currently available, and we have asked relevant articles in the IEEE access journals about the possibility of sharing the datasets, but there has been no response.

2. Insufficient analysis of model complexity and computational cost:
   While the Swin Transformer, along with the HS-FPN and SAM modules, improves performance, the computational complexity and training time of these deep learning models may be high.

Response：Comparison and analysis of model params and theo flops have been added to the experimental section, and corresponding parameter data have been added to the tables.

3. Lack of detail in defect bean classification:
   The paper mentions that the classification of defective beans in the grading process is somewhat vague, and while the authors introduce SCA-recommended categories, the classification of defective beans may still lack precision.

Response：Details of defect categories and corresponding map accuracy and F1 values for each defect type have been added, tables and analyses have been added to the experimental section.

Reviewer #2:

4. The manuscript lacks a clear Data Availability Statement, as PLOS ONE's policy requires. While the authors mention using a proprietary dataset and a publicly available dataset from Kaggle, it is unclear if the proprietary dataset is made fully available without restriction. The authors should explicitly provide access to the data or clarify any restrictions related to its availability. Additionally, the dataset link provided seems to be incorrect or broken and should be reviewed.

Response：Proof of data availability has been added, links to the dataset have been fixed, and the specified pages can now be viewed. The shared and proprietary datasets used in the text are available and the code and some of the datasets have been shared to the web page (https://github.com/tonyalice77/Papers-related-to-swin-transformer-improvements/tree/master) The original files of the datasets are quite large and will be uploaded later, if you have any other questions, please contact us with 690822961@qq.com

5. The use of acronyms (e.g., mAP, SAM, HS-FPN) is consistent, but it would be helpful to define these terms more clearly early on for readers unfamiliar with the specific methods.

Response：Notes have been added for each term where it first appears to help readers better understand the text

6. The authors should present a more comprehensive analysis in discussion part.

Response：The discussion has been supplemented with a more detailed analysis of model performance, types of defective beans, and model interpretability, as well as an outlook on what needs to be added to the model and what can be improved afterward

7. Before submitting your manuscript, I strongly recommend reviewing the formatting to align with PLOS ONE’s submission guidelines. Several formatting issues, such as missing references (e.g., "Error! Reference source not found"), should be corrected. These errors can significantly impact the professionalism and readability of the manuscript. Please refer to the journal's submission guidelines to ensure all requirements are met.

Response：Changes have been made to the manuscript in accordance with the PLOS submission guidelines, such as changing the citation format.
